# Factors associated with a measles outbreak in children admitted at Mahosot Hospital, Vientiane, Laos

**DOI:** 10.1186/1471-2458-7-193

**Published:** 2007-08-04

**Authors:** Mayfong Mayxay, Tiengthong Khomthilat, Phoutthalavanh Souvannasing, Khamphouvanh Phounesavath, Banlieng Vorasane, Sommay Keomany, Phouvieng Douangdala, Khamseng Philavong, Leila Srour, Paul N Newton

**Affiliations:** 1 Division of Post-Graduate studies and Research, Faculty of Medical Sciences, National University of Laos, Vientiane, Lao PDR; 2 Wellcome Trust – Mahosot Hospital – Oxford Tropical Medicine Research Collaboration, Vientiane, Lao PDR; 3 Mother and Child Health Care Centre, Ministry of Health, Lao PDR; 4 Health Frontiers, Vientiane, Lao PDR; 5 Centre for Tropical Medicine, Nuffield Department of Clinical Medicine, Churchill Hospital, University of Oxford, Oxford, UK

## Abstract

**Background:**

In 2002 and 2003 there were large outbreaks of measles in many provinces of Laos, including in Vientiane. We therefore conducted a study to determine risk factors associated with measles amongst children admitted at Mahosot Hospital, Vientiane.

**Methods:**

A retrospective case-control study was conducted in 50 children with clinical measles who were matched by age and sex with 50 healthy children (who had never had a febrile rash) living in the same villages as the cases.

**Results:**

The proportion of children with complete immunizations was significantly lower in the group with clinical measles compared to the controls [13/50 (26%) vs 34/50 (68%), *P *< 0.001). The percentage of children who had received measles vaccine at 9–23 months of age was significantly lower in the group with clinical measles compared to the healthy controls [12/50 (24%) vs 24/50 (48%), *P *= 0.01). The family educational and socio-economic status did not differ significantly (*P *> 0.05) between cases and controls.

**Conclusion:**

These results emphasize the importance of intensification of measles immunization coverage in Laos. The strengthening of campaigns with large, widespread high second dose coverage is likely to be a key measure to prevent further measles outbreaks in Laos (192 words).

## Background

Although great efforts have been exerted by the World Health Organization (WHO) to reduce the public health burden of measles, the disease remains the leading cause of vaccine-preventable child morbidity and mortality worldwide, particularly in developing countries where immunization coverage is low and vitamin A deficiency is common. Globally, more than 20 million measles cases are reported annually with 345,000 deaths in 2005, which represent 50–60% of the estimated 1.6 million deaths attributed to vaccine-preventable diseases of childhood [1-3]. Failure to deliver at least one dose of measles vaccine to all infants remains the main reason for high measles morbidity and mortality as ≥95% coverage is required to interrupt measles transmission [2].

In Laos measles remains an important cause of morbidity and mortality in children under five years of age. Measles outbreaks, confirmed by IgM antibody diagnosis, have been recently reported in many parts of the country [4,5]. At least 2,000–5,000 measles cases, with 20–50 deaths, are annually reported (Centre of National Laboratory and Epidemiology, Vientiane, Laos, unpublished), but this figure is likely to be an underestimate. The Lao national policy for measles immunization is the administration of one dose of measles vaccine to all children aged 9–23 months. The official country-wide report of measles vaccination coverage is usually over 60% [4], with coverage of 68% in 2002 in Vientiane [6]. In 2000, in order to augment the measles immunization coverage, the government began the provision of a "second opportunity" for measles vaccination to children 9 months to 5 years through supplementary campaigns covering the entire country. Recently, there has been uncertainty about the coverage of the measles immunization campaign in Laos [4,7,8]. In 2002, there were large outbreaks of measles in Phongsaly and Vientiane provinces, where immunization campaigns were conducted in 2000 and 2001, respectively [7]. Six hundred and thirty-six measles cases (43% children were under 5 years old) including 11 deaths were reported in Phongsaly, and 219 cases (74% under 5 years) were reported in Vientiane. In late 2002 and early 2003, measles epidemics were reported from at least 6 provinces of Laos and in the first 3 months of 2003 more than 150 patients with measles were admitted to the paediatric wards of Mahosot Hospital in Vientiane. In order to determine the factors associated with the disease outbreak and provide information for future disease prevention planning, we conducted a retrospective case-control study comparing potential risk factors between children with and without measles who were admitted at Mahosot Hospital.

## Methods

A case-control study was conducted on the paediatric wards of Mahosot Hospital (a 365 bed-referral hospital). Children aged ≤ 15 years old who were clinically diagnosed as having measles (defined as 'clinically confirmed cases' i.e. fever, maculopapular rash (non-vesicular), cough, and coryza or conjunctivitis [9])by the ward paediatricians between January and March 2003 were included in the study provided that their hospital charts included details of symptoms and signs and demographic data, their houses were ≤ 50 km (patients living more distant were difficult to trace) from the hospital and their parents gave oral informed consent to the study. The demographic and clinical data were extracted from their hospital charts and were matched for sex and age (± 1 year) with healthy children who had no history of febrile rash and who lived in the same villages as those with measles attacks. The villages were visited ~6 weeks after patient admission and the children were examined and their parents interviewed. A standard questionnaire was designed in order to collect all information thought to be associated with measles outbreaks, including childrens' feeding, nutritional (including food avoidance behaviour) and immunization status, parents' educational level and socio-economic status and preventive measures taken when measles occurred in the family. Immunization histories were checked against the immunization cards of the children. Nutritional status was assessed by height-for-age Z scores (HAZ) [10]. The study was performed according to the World Medical Association Declaration of Helsinki (52^nd ^General Assembly, 2000). Data were analyzed using SPSS version 8.0 (SPSS, Chicago, Il, USA) and WHO Anthro2005 software [11]. Comparisons between groups were made using paired or unpaired Student's t and Chi-square tests as appropriate.

## Results

### Children

Fifty children who had clinical measles (cases) were matched for sex and age with 50 children who were healthy, without a history of febrile rash (controls) (Table 1). There was no significant difference in the feeding method or duration between the children who had measles and the control children (Table 1). Fifty percent of all children stopped breastfeeding at the age of 13–18 months. The median (range) admission HAZ score was -1.87 (-4.34 to +2.94); 17 of 46 children (37%) with available data had HAZ scores of <-2.0, indicating severe stunting. The percentage of children who had received vitamin A supplementation within 6 months was significantly higher in the group with measles [30/50 (60%)] compared to the control group [19/50 (38%)] (*P *< 0.001), as most children with measles were given vitamin A during hospital admission.

**Table 1:** Demographic, clinical, feeding and immunization characteristics of children at interview (data shown as number (%) unless indicated)

Variables	Cases (n = 50)	Controls (n = 50)	*P*-value
Age (year) ^a^	5.8 (4.6–7.0)	5.8 (4.7–6.9)	-
Sex: Male/Female	20/30	20/30	-
No. schooling/kindergarten	31 (62%)	25 (50%)	0.23
Breastfed	46 (92%)	47 (94%)	1.00
Colostrums-fed at birth	42 (87%)	45 (92%)	0.65
Exclusive breastfed up to 6 months	35 (70%)	35 (70%)	1.00
Age at which supplementary food started (months) ^a^	3.5 (1.8–4.6)	3.6 (1.7–5.1)	0.96
Overall complete immunization	13 (26%)	34 (68%)	<0.001
Measles immunization at 9–23 months	12 (24%)	24 (48%)	0.01
One dose of BCG at birth	39 (78%)	46 (92%)	0.05
Three doses of polio immunization	19 (38%)	32 (64%)	0.009
Three doses of DPT	18 (36%)	32 (64%)	0.005

^a ^Mean (95% CI)

DPT = diphtheria, pertussis and tetanus immunization

The proportion of children with immunization cards was similar between cases and controls [18/50 (36%) vs 14/50 (28%), *P *= 0.32]. The overall immunization and measles immunization coverage were significantly lower in the children who had measles compared to those who had not [13/50 (26%) and 12/50 (24%) vs 34/50 (68%) and 24/50 (48%); *P *< 0.001 and *P *= 0.01, respectively] (Table 1). The most frequent reason the parents gave for not taking their children to have vaccination was fever following vaccination (30%), inaccessibility of medical staff to the village (22%), unawareness as to where to have their children immunized (14%) and other reasons (34%).

### Parents

A total of 100 parents (23 fathers and 77 mothers) of 50 cases and 50 controls were interviewed (Table 2). Eighty-six percent were lowland Lao (Lao Lum) and 87% were Buddhists. The overall mean parent (95%CI) age was 33.8 (30.5–35.4) years and there was no significant difference between the two groups. The most frequent occupations of mothers and fathers were traders (27%) and construction workers (32%), respectively. Sixty-six percent of all the parents practiced food avoidance behaviour for their children when the child became sick. The proportion of parents who practiced food avoidance behaviour was higher in the group with measles in comparison to those without measles but the difference was not statistically significant [37/50 (74%) vs 29/50 (58%), *P *= 0.06]. Fruits were the most common items that the parents did not allow their children to eat when they are sick (71%). The parents' knowledge of measles was very low; only 9% in both groups knew that measles can be transmitted by inhalation and only 18% knew that the disease could be prevented by vaccination. The proportion of parents who stated that measles is dangerous was significantly higher in the parents of cases compared to those of controls [41/50 (82%) vs 31/50 (62%), *P *= 0.03). The proportion of parents who would isolate the patient with measles from other family members was 34% in the controls and 20% in the measles group (*P *= 0.11). The house area, the number of bedrooms and the number of neighboring houses were similar between the cases and controls. However, the mean (95%CI) number of family members per household was significantly higher in the group with measles than in the control group [7.4 (6.9–8.3) vs 6.1 (5.4–7.5), *P *= 0.01).

**Table 2:** Demographic data of all interviewees or patients' guardians at interview (datashown as number (%) unless indicated)

Variables	Cases (n = 50)	Controls (n = 50)	*P*-value
Age (year) ^a^	35.0 (32.2–37.4)	32.6 (30.2–34.8)	0.19
Sex: Male/Female	15/35	8/42	0.09
Ethnicity:			
Lao Loom	43 (86%)	43 (86%)	1.00
Lao Sung	5 (10%)	7 (14%)	-
Lao Theung	2 (4%)	0	-
Know at least one signs or symptoms of measles	33 (66%)	31 (62%)	0.68
Consider measles as dangerous	41 (82%)	31 (62%)	0.03
Would isolate patients with measles	10 (20%)	17 (34%)	0.11

^a ^Mean (95% CI)

## Discussion

We attempted to describe factors associated with a measles outbreak in Laos, finding that feeding and nutritional practices, family socio-economic and parents' educational status were not significantly different between the groups of children with and without measles. In contrast, the proportion of overall immunizations and measles immunizations were significantly lower in the children who had measles compared to those who had not, suggesting that poor immunization coverage plays a crucial role in measles outbreaks in Laos. Cases were more likely to be from a larger family than controls. Lower frequencies of measles, mumps and rubella vaccination have been described in the youngest children in UK families [12]. The study is limited by the lack of laboratory confirmation of measles cases, the relatively low proportion of children with immunisation cards, that the cases were only those admitted to hospital and possible recall bias.

The one-dose measles immunization coverage among all children (9–23 months) in this study was only 36%. This figure is consistent with the measles immunization coverage of only 34.6% of 185 measles cases (confirmed by IgM antibody testing) reported during an outbreak in four Lao provinces [4]. However, the official report of measles vaccination coverage in Vientiane Prefecture in 2002 was 68% [6], and the nationally official report throughout the country was 65–73% during 1996–1999 [4]. A measles outbreak in an orphanage in Bangkok, Thailand, in 2000 was also associated with a low coverage of measles immunization of only 45% of infants over 9 months [13]. Vitamin A coverage in hospital for the measles patients was also low at 60% and the avoidance of fruit during and after measles may exacerbate vitamin A deficiency.

The Lao national policy is for one dose measles immunization at the age of 9–23 months for all children. Measles can occur in children before the age of measles immunization, as demonstrated in this study in which six percent of study children had measles before they were 9 months. Although maternal-derived immunity against measles in infants significantly drops before the age of six months, infants of this age mount a lower humoral immune response than those aged ≥9 months [14,15]. Therefore, whether reducing the age of first measles immunization would be efficacious remains unclear.

One dose measles vaccination at the age of 9–23 months may not be able to protect children from measles. Indeed, approximately one quarter of the children who had one-dose measles vaccines in the present study developed measles, perhaps because of failures in the cold chain reducing vaccine efficacy [4]. Single-dose measles vaccinated children have been reported to develop measles [16,17]. Therefore, even if the one dose measles immunization coverage in Laos reaches ≥95%, a substantial proportion of Lao children may still develop measles. The standard regimen of measles immunization (usually in combination with mumps and rubella) in developed countries is at least two doses; the first at 12 – 15 months of age with a second dose at 4–6 years or at 11–12 years of age [9,18,19]. The Lao government has been providing a "second opportunity" for measles vaccination through supplementary campaigns covering the entire country. As suggested by Kuroiwa [7] a "third opportunity" may be a good way to augment the coverage as high as possible in order to interrupt virus transmission. A pre-school booster when the first measles vaccination coverage reaches >80% should be considered.

Fever following vaccination may be an important factor contributed to the low immunization coverage. More research is needed as to the reasons children are not vaccinated and given vitamin A and on the importance of food avoidance behaviour in reducing vitamin A intake at such a vulnerable time.

## Conclusion

The enhancement of country-wide, regular sustainable vaccination, with the implementation of second doses at high coverage, will be crucial in reducing the incidence of measles in Laos. Health education on the dangers of measles and the safety of immunization should be provided to the mothers and fathers of Lao children.

## Competing interests

The author(s) declare that they have no competing interests.

## Authors' contributions

MM, LS, TK, PS, KP, BV, SK, PD & KP designed the study and collected the data; MM, LS & PN analyzed the data and drafted the paper. All authors read and approved the final manuscript.

## Pre-publication history

The pre-publication history for this paper can be accessed here:


